# Super-Resolution Imaging of Plasma Membrane Proteins with Click Chemistry

**DOI:** 10.3389/fcell.2016.00098

**Published:** 2016-09-09

**Authors:** Pablo Mateos-Gil, Sebastian Letschert, Sören Doose, Markus Sauer

**Affiliations:** Department of Biotechnology and Biophysics, Julius Maximilian University of WürzburgWürzburg, Germany

**Keywords:** super-resolution fluorescence microscopy, localization microscopy, *d*STORM, plasma membrane organization, click chemistry, protein clusters

## Abstract

Besides its function as a passive cell wall, the plasma membrane (PM) serves as a platform for different physiological processes such as signal transduction and cell adhesion, determining the ability of cells to communicate with the exterior, and form tissues. Therefore, the spatial distribution of PM components, and the molecular mechanisms underlying it, have important implications in various biological fields including cell development, neurobiology, and immunology. The existence of confined compartments in the plasma membrane that vary on many length scales from protein multimers to micrometer-size domains with different protein and lipid composition is today beyond all questions. As much as the physiology of cells is controlled by the spatial organization of PM components, the study of distribution, size, and composition remains challenging. Visualization of the molecular distribution of PM components has been impeded mainly due to two problems: the specific labeling of lipids and proteins without perturbing their native distribution and the diffraction-limit of fluorescence microscopy restricting the resolution to about half the wavelength of light. Here, we present a bioorthogonal chemical reporter strategy based on click chemistry and metabolic labeling for efficient and specific visualization of PM proteins and glycans with organic fluorophores in combination with super-resolution fluorescence imaging by *direct* stochastic optical reconstruction microscopy (*d*STORM) with single-molecule sensitivity.

## Introduction

The plasma membrane in eukaryotes is involved in several cell functions such as tissue formation, signal transduction, cell adhesion, and immune response. Although much evidence suggests that the spatial arrangement of its different components, i.e., membrane proteins and lipids, determines the functionality of the PM of eukaryotic cells, the precise molecular architecture remains unclear. Our current view of the cell membrane goes beyond the “fluid mosaic model,” proposed more than 40 years ago by *Singer and Nicolson*, where proteins freely diffuse in a homogeneous sea of lipids (Singer and Nicolson, 1972). In contrast, a hierarchical subcompartmentalization, where proteins are transiently trapped in lipid rafts and actin-cytoskeleton associated corrals, has been hypothesized (Kusumi et al., 2012). Dynamic data obtained by ultra-fast single particle tracking has shown reduced diffusion behavior and hoping events of different membrane proteins suggesting the presence of protein nanodomains (Kusumi et al., 2005). The predicted size of these nanoclusters is in the order of a few tens to a few hundreds of nanometers, dependent on the cell type, protein, or lipid. However, until now two obstacles impede the exploitation of quantitative data about the architecture of membrane-associated glycoproteins: selective and efficient labeling of membrane components and the resolution limit of optical microscopy.

During the last decade, the advent of far-field super-resolution microscopy methods, such as stochastic optical reconstruction microscopy (STORM) (Rust et al., 2006), *direct*STORM (Heilemann et al., 2008; van de Linde et al., 2011), photoactivated light microscopy (PALM) (Betzig et al., 2006), fluorescence PALM (Hess et al., 2006), stimulated emission depletion microscopy (STED) (Klar et al., 2000), ground state depletion microscopy (GSD) (Bretschneider et al., 2007), and structured illumination microscopy (SIM) (Gustafsson, 2000), has overcome this limitation. The application of these techniques revealed the existence of PM clusters with a typical size of ~80 nm for various PM proteins (Kittel et al., 2006; Sieber et al., 2007; Williamson et al., 2011; Bar-On et al., 2012; Rossy et al., 2013). However, probing weather protein subcompartmentalization is a universal feature of PMs is still challenging. To this aim, methods devoted to stain, and visualize simultaneously a large population of PM proteins are required. Electron microscopy using immunogold labeling on isolated plasma membrane sheets revealed the existence of highly dense patches containing different membrane proteins (Lillemeier et al., 2006). More recently, the introduction of a bioorthogonal chemical reporter strategy, based on metabolic labeling and click chemistry, allowed the direct visualization of different membrane components by super-resolution microscopy (Letschert et al., 2014; Saka et al., 2014). This approach exploits the ability of the endogenous metabolic cellular machinery to recognize different metabolic surrogates containing small reactive chemical modifications ready to be conjugated with fluorophores. Non-natural methionine analogs, containing an azide, or an alkyne group, are recognized by the methionyl-tRNA synthetase and co-translationally incorporated into nascent proteins (Tom Dieck et al., 2012). On the other hand, non-native monosaccharide precursors can be used to introduce similar chemical groups into glycoproteins as post-translational modifications (Laughlin and Bertozzi, 2009a). Thus, click chemistry represents a direct labeling method for the visualization of different PM components.

Here, we report an efficient method to visualize PM proteins stained via metabolic labeling and click chemistry by super-resolution imaging with virtually molecular resolution. In particular, we present two procedures enabling quantitative super-resolution imaging of PM components on two different time-scales. First, we use L-azidohomoalanine (L-AHA), a non-natural methionine analog that is incorporated into newly synthesized proteins, typically within few hours. Second, we use peracetylated N-azidoacetylgalactosamine (Ac_4_GalNAz) as a non-native monosaccharide incorporated into membrane-associated glycoproteins during 2 days of incubation. For fluorescence labeling, we compare two click chemistry reactions, copper-catalyzed azide-alkyne cycloaddition (CuAAC), and copper-free strain-promoted azide- alkyne cycloaddition (SPAAC), with regard to labeling efficiency. For fluorescence imaging with subdiffraction-resolution, we use single-molecule localization microscopy based on photoswitching of standard fluorophores, i.e., *direct* stochastic optical reconstruction microscopy (*d*STORM) (Heilemann et al., 2008; van de Linde et al., 2011). Furthermore, we describe localization microscopy based methods to determine quantitative information on density and spatial distribution of membrane proteins such as Ripley's K function. In addition, we highlight advantages of the method and limitations that might give rise to the appearance of artificial membrane clusters. Our data indicate that high emitter densities can be achieved of both apical and basal membrane components. Inhomogeneous distributions of PM proteins or glycans are revealed, especially in two-dimensional projections of intrinsically three-dimensional (3D) structures such as filopodia and overlapping membranes. More importantly, labeled vesicles located in close proximity to the PM can be misleadingly interpreted as clusters in two-dimensional super-resolution images. A certain degree of deviation from complete spatial randomness in PM proteins was found by Ripley's K function analysis.

## Materials

### Cell culture and maintenance

1. Cell line and growth media: Adherent cell line growth in appropriate culture media. In this case, we use a human osteosarcoma (U2OS) cell line in standard growth media (cDMEM: Dulbecco's modified Eagle's HAM's F12 media supplemented with 10% (v/v) fetal calf serum (FCS), 4 mM glutamine, 100 U/L penicillin, and 0.1 mg/mL streptomycin).
2. Cell culture and maintenance: T25-culture flasks (Greiner Bio-One). Cell culture incubator maintained in humidified atmosphere at 5% CO_2_ and 37°C. Phosphate-buffered saline (PBS), Hank's balance salt solution (HBSS), and accutase solution.
3. Cell preparation for metabolic labeling and fluorescence imaging: 8 well Lab-Tek chamber slides (Nunc, Thermo Fisher Scientific).

### Metabolic labeling with azido unnatural amino acid AHA

1. Metabolic labeling media: Methionine free media (MFM: Dulbecco's modified Eagle's HAM's F12, with 10% FCS, 4 mM glutamine, 100 U/L penicillin, and 0.1 mg/mL streptomycin, without methionine).
2. Azido methionine analog: L-azidohomoalanine (L-AHA) (Jena Bioscience) stored as powder at 4°C.
3. Protein synthesis inhibitors: Anisomycin (Sigma-Aldrich) 10 mg/mL stock solution in dimethyl sulfoxide (DMSO)

### Metabolic labeling with peracetylated azido modified monosaccharides.

1. Metabolic labeling media: Standard growth media (cDMEM) supplemented as described in cell culture and maintenance.
2. Azido modified monosaccharides: N-azidoacetylgalactosamine (Ac_4_GalNAz) (Invitrogen). Stock solutions were prepared at 25 mM in dimethyl sulfoxide (DMSO) and stored at −20°C up to 12 months.

Alternatively N-azidoacetylmannosamine (Ac_4_ManNAz) and N-azidoacetylglucosamine (Ac_4_GlcNAz) can be used

### Copper-catalyzed azide-alkyne cycloaddition (CuAAC)

1. Staining solution additives: Copper sulfate (CuSO_4_), copper ligand Tris(3-hydroxypropyltriazolyl-methyl)amine (THPTA), and sodium ascorbate (Sigma-Aldrich).
2. Stock solutions of 2 mM CuSO_4_ and 10 mM THPTA in MiliQ water stored at −20°C. 100 mM sodium ascorbate in MiliQ water freshly prepared.
3. Alkyne-tagged fluorophore: 2 mM stock solution of Alexa Fluor 647 alkyne (Thermo Fischer Scientific) in DMSO stored at −20°C up to 12 months.

### Strain-promoted azide-alkyne cycloaddition (Spaac)

1. DBCO-tagged fluorophore: 2 mM stock solution of Cy5 DBCO (Sigma-Aldrich) in DMSO stored at −20°C up to 12 months.

### Super-resolution imaging with *d*STORM

1. Setup: We used a custom-made setup based on an inverted commercial microscope (IX71; Olympus) equipped with an oil-immersion objective (60x, NA 1.45; Olympus), and a nosepiece stage (IX2-NPS; Olympus) to prevent focus-drift during image acquisition. A 641-nm diode laser (Cube 640–100C; Coherent), spectrally cleaned-up with a band-pass filter (BrightLine 642/10, Semrock), was used for excitation of Cy5 and AF-647. Additionally, two lenses and a mirror, coupled to a translation stage, were used to focus the laser beam on the back focal plane of the objective and switching between different illumination modes, i.e., epi, low-angle/highly inclined and laminated light optical sheet (HILO), and total internal reflection illumination (TIR) (Sharonov and Hochstrasser, 2007; Tokunaga et al., 2008; van de Linde et al., 2011). Fluorescence emission of Cy5 and AF-647 were collected with the same objective, separated from excitation light by a dichroic beamsplitter (560/659, Semrock), filtered with appropriate band- and long-pass filters (BrightLine 697/75 and RazorEdge 647, Semrock), and projected on an EMCCD camera (Ixon DU897, Andor Technology). Additional lenses were placed into the detection path to generate a final pixel size of 134 nm.
2. Switching buffer: PBS buffer containing 100 mM β-mercaptoethylamine (MEA, Sigma-Aldrich) and an oxygen scavenger system (2% (w/v) glucose, 4 U/mL glucose oxidase and 80 U/mL catalase) adjusted to pH 7.4.
3. *d*STORM image reconstruction: Open source software for single-molecule localizations and super-resolution image reconstruction rapi*d*STORM 3.3 (Wolter et al., 2010, 2012).

### Quantitative analysis

For quantitative analysis of generated localization data based on XY coordinates lists, customized algorithms implemented with programing languages such as Python (available at http://www.python.org), and Mathematica (Wolfram Research Inc., Champaing, Il, USA) were used.

## Methods

### Background

Since the development of the *Staudinger-Bertozzi ligation* between azides and phosphines in 2000 (Saxon and Bertozzi, 2000), bioorthogonal “*click chemistry”* reactions allowed the visualization of different biomolecules (e.g., proteins, glycans, lipids, and nucleic acids) in cultured cells, tissues, and living organisms (Sletten and Bertozzi, 2009). To this aim, one functional group (the label) is introduced into the biomolecule of interest followed by exogenous addition of fluorophores bearing the reactive partner (the probe). For example, unnatural amino acids and monosaccharides containing an azide group can be used as metabolic surrogates of their native counterparts to visualize proteins and glycoproteins as well as glycolipids (Laughlin and Bertozzi, 2009a; Tom Dieck et al., 2012).

Two different approaches have been used successfully to introduce amino acid analogs into proteins: (i) genetic encoding, i.e., site-specific modification, and (ii) metabolic labeling, i.e., residue-specific modification. Whereas, the first method introduces unnatural amino acids into one particular protein, the second method allows labeling of a wide part of the proteome replacing a native amino acid (e.g., methionine) by its non-natural analog (e.g., L-azidohomoalanine, L-AHA). Due to its structural similarity, L-AHA is recognized and tolerated by the methionyl-tRNA synthetase (MetRS), and incorporated into newly synthesized proteins co-translationally in a residue-specific manner. Alternatively, azido sugars (e.g., peracetylated N-azidoacetylgalactosamine Ac_4_GalNAz, N-azidoacetylmanosamine Ac_4_ManNAz, and N-azidoacetylglucosamine Ac_4_GlcNAz), can be incorporated into different types of glycoproteins and glycolipids (Laughlin et al., 2006; Laughlin and Bertozzi, 2009a). Upon cellular uptake and deacetylation, Ac_4_GalNAz, Ac_4_ManNAz, and Ac_4_GlcNAz are converted into activated sugars, recognized by the glycan biosynthetic machinery, and incorporated into sialic acids and mucin-type O-linked glycans, as well as into O-GlcNAc-modified proteins. After metabolic incorporation of amino acids and monosaccharide surrogates, the azide groups introduced into newly synthesized proteins and glycans can be conjugated with alkyne fluorophores via azide-alkyne cycloaddition allowing their direct visualization.

Originally, the classic reaction between terminal alkynes and azides was shown to be efficiently catalyzed by copper(I) at room temperature enabling it to proceed within minutes under physiological conditions, opening the door for biological applications (Rostovtsev et al., 2002; Tornøe et al., 2002). Since then, this reaction, now termed as the Cu(I)-catalyzed azide-alkyne cycloaddition (CuAAC), has been used to visualize different metabolically labeled biomolecules (Sletten and Bertozzi, 2009). However, due to Cu(I) toxicity fluorescent staining by CuAAC has been restricted to fixed cells. To overcome this problem, two alternative strategies have been developed. In 2004, it was shown that azide-alkyne cycloaddition can be strain-promoted in the absence of copper(I) using cyclooctynes (Agard et al., 2004). Since then, different cyclooctyne molecules with enhanced efficiency have been developed for copper-free strain-promoted azide-alkyne cycloaddition (SPAAC) (Jewett and Bertozzi, 2010; Debets et al., 2011). On the other hand, the optimization of the CuAAC, by means of copper(I) ligands and further additives in the reaction buffer, preserves cell viability while live staining. For example, the use of THPTA in addition to sodium ascorbate allow efficient CuAAC bioconjugation within 5 min with low copper concentrations (e.g., 50 μM) minimizing Cu(I) toxic effects (Hong et al., 2009, 2010).

Standard fluorescence microscopy, combined with metabolic labeling and click chemistry, has been used extensively to visualize both proteins and membrane-associated glycoconjugates within different cellular contexts. For example, newly synthesized proteins have been imaged in mammalian cells and rat hippocampal neurons (Dieterich et al., 2006, 2010; Beatty and Tirrell, 2008), and different glycan populations in culture cells (Baskin et al., 2007), developing zebrafish embryos (Laughlin et al., 2008), and living *C. elegans* (Laughlin and Bertozzi, 2009b). Remarkably, these studies demonstrated the versatility of metabolic labeling for temporal profiling of dynamic changes in large protein populations and glycans. More recently, the same chemical reporter strategy has allowed direct visualization of different membrane components by super-resolution microscopy (Letschert et al., 2014; Saka et al., 2014). Stimulated emission depletion (STED) was used to image unnatural amino acids incorporated into membrane proteins in monkey kidney cell line COS-7, demonstrating protein confinement with reduced diffusion dynamics (Saka et al., 2014). On the other hand, *d*STORM was used to visualize different glycan types, including glycoproteins, after metabolic labeling with Ac_4_GalNAz, Ac_4_ManNAz, and Ac_4_GlcNAz in human osteosarcoma (U2OS) and neuroblastome (SK-N-MC) cells (Letschert et al., 2014). Moreover, due to its ability for single-molecule detection and position determination *d*STORM measurements provided quantitative estimates of molecular densities and spatial distributions of membrane-associated glycoconjugates.

### Protocols

**Figure 1:**
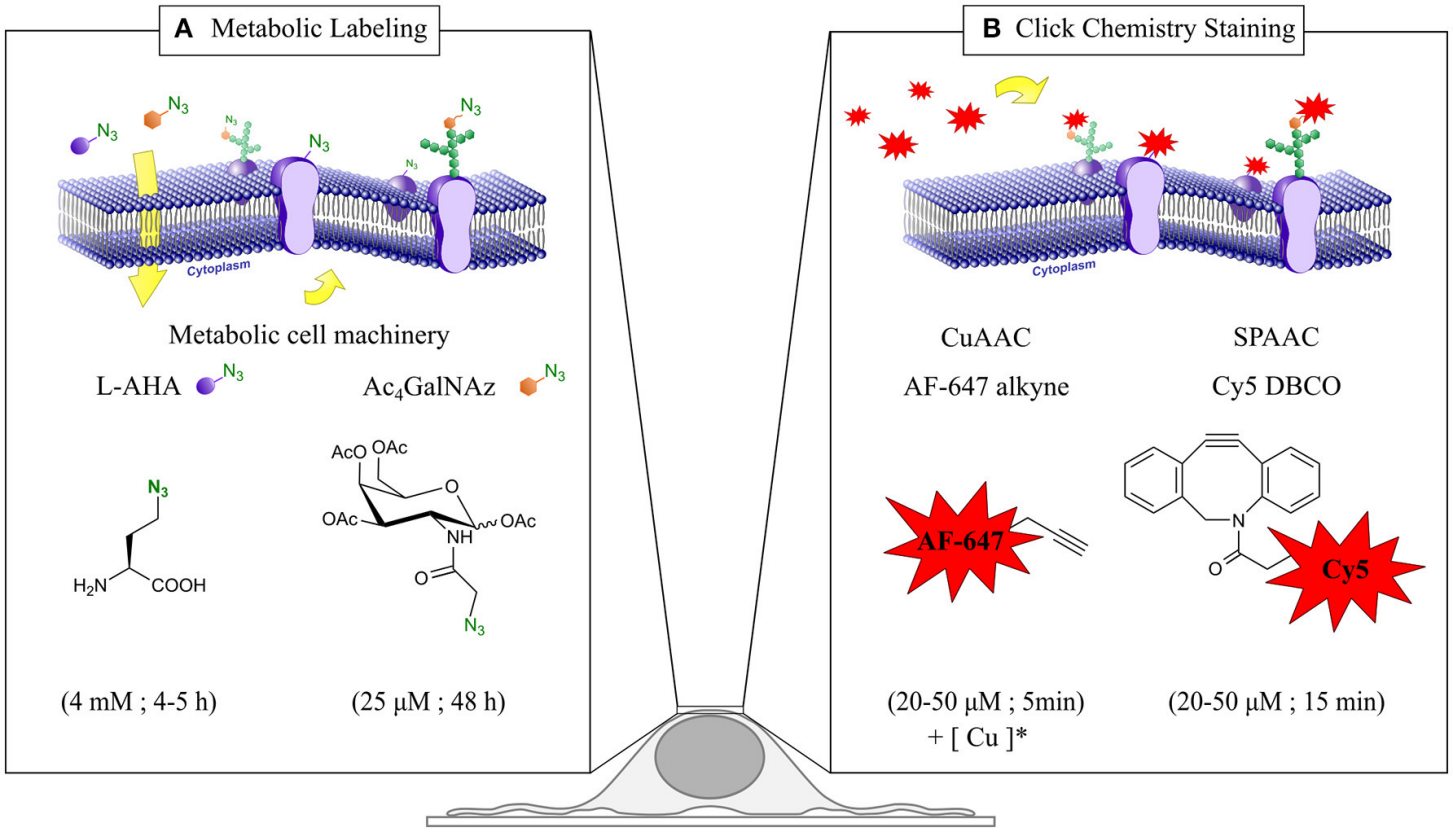
**Chemical reporter strategy based in metabolic labeling and click chemistry for ***d***STORM quantitative imaging of plasma membrane (PM) proteins**. **(A)** Incorporation of the metabolic surrogates L-azidohomoalanine (L-AHA) and N-Azidoacetylgalactosamine (Ac_4_GalNAz) into newly synthesized proteins. Upon cellular uptake, L-AHA and Ac_4_GalNAz are recognized by the endogenous cell machinery, and incorporated co-translationally and post-translationally into PM proteins and glycoproteins respectively. Typical incubation time and surrogate concentration for each metabolic labeling scheme are indicated at the bottom. **(B)** Click chemistry staining of PM proteins. After metabolic labeling, PM proteins bearing an azido group are stained with Alexa Fluor 647 alkyne and Cy5 DBCO via copper-catalyzed (CuAAC) and copper-free azide-alkyne cycloadditions (SPAAC) respectively. Typical incubation time and fluorophore concentration for each click chemistry reaction are indicated at the bottom. (^*^) Staining solution for CuAAC reaction (50 μM CuSO_4_, 250 μM THPTA, 2.5 μM sodium ascorbate, and the desired amount of Alexa Fluor 647 alkyne in PBS).

In this section we provide protocols to combine metabolic labeling and fluorescent staining via click chemistry for super-resolution imaging with *d*STORM of membrane proteins with single-molecule sensitivity. The method comprises four steps:

**Step 1. Metabolic labeling with azido surrogates**, i.e., with L-azidohomoalanine (L-AHA) and peracetylated N-azidoacetylgalactosamine (Ac_4_GalNAz) (Figure 1A).
**Step 2. Click chemistry fluorescent live staining** via copper-catalyzed (CuAAC) and copper-free strain-promoted azide-alkyne cycloadditions (SPAAC) (Figure 1B).
**Step 3. Localization based super-resolution imaging with *d*STORM**. Image acquisition and reconstruction, identification of two-dimensional projections of three-dimensional cell structures, and labeling efficiency estimation.
**Step 4. Quantitative analysis**. Estimation of detected molecular densities using reference samples, and clustering analysis by Ripley's K function.

### Step 1- metabolic labeling with azido surrogates

#### Protocol 1a: metabolic labeling with azido methionine analogs (L-azidohomoalanine, L-AHA)

1. Cell culture and maintenance: Choose an appropriate cell line, e.g., human osteosarcoma (U2OS) cells, as a model system of adherent mammalian cells. Maintain the cells at 37°C in 5% CO_2_ water-saturated atmosphere in growth culture medium (cDMEM).
  For gentler detachment of cells from T25-culture flasks incubation with accutase for 5 min is preferred rather than trypsine/EDTA treatment.
2. Azido amino acid incubation: Detach cells from culture flask by incubating with accutase for 5 min, count them and seed them in LabTek 8 well chambers at 1.2 × 10^4^ final concentration per well in cDMEM growth media, and let them grow in the cell incubator for 48–72 h at 37°C and 5% CO_2_ water saturated atmosphere until 80–90% confluency. Previous to L-AHA incubation, exchange growth medium with prewarmed HBSS, and incubate cells at 37°C during 50 min to deplete the cellular reservoirs of endogenous methionine. During this time prepare a fresh solution of 4 mM L-AHA in methionine-free medium (MFM) and prewarm it. Replace HBSS with AHA solution and incubate cells at 37°C and 5% CO_2_ water saturated atmosphere for the desired time, e.g., 4–5 h.
  Control samples can be prepared incubating AHA in the presence of protein synthesis inhibitor such as anisomycin at 40 μM final concentration to evaluate fluorescent background (Figure S1).

#### Protocol 1b: metabolic labeling with azido sugars (N-azidoacetylgalactosamine, Ac_4_GalNAz)

1. Cell culture and maintenance: follow the same procedure as describe above.
2. Azido sugar incubation: After accutase incubation seed the cells onto 8 wells LabTek chamber at a final concentration of 1.2 × 10^4^ cells per well. Add Ac_4_GalNAz at 25 μM final concentration. Incubate cells at 37°C and 5% CO_2_ water saturated atmosphere for 48 h before fluorescence staining and fixation.
  Control cells can be prepared in absence of azido sugars to evaluate fluorescence background (Figure S1).

### Step 2- fluorescence live staining via CuAAC and Spaac

#### Protocol 2a: copper catalyzed azide-alkyne cycloaddition (CuAAC)

1. Preparation of optimal staining solution (50 μM CuSO_4_, 250 μM THPTA, 2.5 μM sodium ascorbate, and the desired amount of Alexa Fluor 647 alkyne in PBS): For one LabTek well (final volume 200 μl). Premix 5 μl of 2 mM CuSO_4_ with 5 μl of 10 mM THPTA stock solution. After 5 min add 5 μl of 100 mM sodium ascorbate freshly prepared stock solution in MiliQ water. Add appropriate volume of PBS and Alexa Fluor 647 depending on the desired final concentration of fluorophore. Vortex at high speed for few seconds.
  Further details in the use of copper ligands and sodium ascorbate for optimal CuAAC bioconjugation can be found elsewhere (Hong et al., 2009).
2. Fluorophore incubation: Immediately after removing the LabTek from incubator, wash cells once with prewarmed PBS and incubate them with staining solution for 5 min protected from light at room temperature. Then, wash cells three times gently with PBS and fixate them in PBS solution containing 4% formaldehyde and 0.2% glutaraldehyde respectively. Finally, wash cells three times with PBS and store them at 4°C in PBS containing sodium azide 0.2% (w/v).
  Strong fixation over long times (e.g., 1 h) in the presence of glutaraldehyde is required to minimized lateral mobility of membrane proteins (Tanaka et al., 2010).

#### Protocol 2b: copper-free strain-promoted azide-alkyne cycloaddition (SPAAC)

1. Staining solution: Dilute Cy5 DBCO in HBSS at desired concentration without any further additives.
  To avoid cellular stress, HBSS is preferred to PBS due to longer fluorophore incubation times.
2. Fluorophore incubation: Proceed as in point 2 of protocol 2a, i.e., wash the cells once with prewarmed PBS, exchange PBS with staining solution with desired fluorophore concentration, and incubate for 15 min instead of 5 min, wash cells three times with PBS, add fixation solution for 1 h, wash three times, and store cells at 4°C in PBS with 0.2% of sodium azide.

### Step 3- localization based super-resolution imaging with *d*storm

#### Protocol 3: *d*STORM super-resolution imaging

1. Photoswitching buffer preparation: Prior to imaging, dissolve β-mercaptoethylamine (MEA) in PBS and keep the MEA powder reagent under argon atmosphere to avoid oxidation. Thaw stock aliquots of glucose, glucose oxidase and catalase for the oxygen scavenger system. Mix all the reagents to final concentrations of 100 mM MEA, 2% (w/v) glucose, 4 U/mL glucose oxidase and 80 U/mL catalase. Finally adjust the pH to 7.4 with 5 M KOH solution.
2. Preparing cells for *d*STORM imaging: Exchange storing buffer with switching buffer (1.1 mL per well) and seal the LabTek with a coverslip to reduce uptake of atmospheric oxygen. Finally mount the LabTek onto the oil immersed inverted objective of the microscope.
3. Measuring *d*STORM image stack: First, localize and position cell of interest at low intensities. Then, increase the irradiation intensity, e.g., 5 kW/cm^2^, to induce rapid transition of the fluorophores to their non-fluorescent off-state. Before image acquisition, exchange the illumination mode from TIRF, to epi-fluorescence and then back to TIRF to maximize the conversion of out-of-focus fluorophores to the dark state. Wait until all molecules in the field of view blink properly, typically 60 s, and start recording an image stack with the desired length and frame rate, e.g., 20,000 frames at 66 Hz (15 ms exposure time per frame).
  High irradiation intensities are crucial while measuring areas with high fluorophore densities to prevent artifacts due to overlapping of single emitter.
4. Reconstruction of super-resolution image with rapi*d*STORM: Set desired values of the minimum intensity threshold for single-molecule localization and the pixel-size of the super resolution image, e.g., 1000 photons and 10 nm respectively.
5. Identification of 2D-projections of 3D cell structures: Image consecutively the region of interest with slightly shifted (0.5–1 μm) focal planes into the cytosol.
6. Estimation of labeling efficiency: Titrate fluorophore concentration for desired fixed metabolic labeling conditions. Calculate localization density using a sliding window analysis (diameter = 1 μm, step = 100 nm). To prevent contribution from overlapping membrane structures measure localization density in regions under the nucleus.

### Step 4- quantitative analysis of molecular densities and spatial distribution at the nanoscale.

#### Protocol 4: estimation of detected molecular densities of membrane proteins and glycans.

1. Preparing reference samples: To ensure detection of single and well isolated fluorophores decrease the labeling density to <20 localizations per μm^2^ by adjusting the fluorophore concentration to <0.1 μM. Perform *d*STORM reference measurements using the same optical and chemical conditions, i.e., laser irradiation intensity, buffer composition and TIRF angle, as for non-diluted samples.
  Grouping localizations from isolated fluorophores: Group all localizations within a certain radius detected along the whole image stack (20,000 frames), e.g., by applying a Kalman tracking routine as implemented in rapi*d*STORM. Allow the tracking algorithm to group localizations with maximum temporal separation equal to stack length within a defined area specified by the given tracking radius. To confirm the detection of single spots vary the tracking radius from 1 to 160 nm.
2. Estimation of detected molecular densities: Plot the average track length versus the tracking radius and use the saturation level of the curve as a conversion factor reflecting the number of localizations detected per isolated fluorophore. In addition, align all the localizations within tracks with length >2 to their center of mass. Calculate the experimental precision by fitting the spatial distribution to a Gauss function.
3. Computation of Ripley's h function: We computed what we call Ripley's h function *h*(*d*) as function of distance *d* following the standard definition for Ripley's k function (Ripley, 1977) and applying an established transformation (Kiskowski et al., 2009) allowing simple optical inspection since *h*(*d*) is equal to zero for all *d* in the case of a spatially homogeneous point process (complete spatial randomness):
  (1) $$\begin{array}{l} h\left(d\right)=\sqrt{\frac{A\sum_{i=1}^{n}\sum_{j=1}^{m}k\left(i,j\right)}{\pi m(n\;-\;1)}}-d \end{array}$$
  where *d* is a distance, *A* is the area of the region containing all localizations, *n* is the total number of localizations, *m* is the number of test localizations in a random subset of localizations, and *k*(*i,j*) is a weight defined as:
  (2) $$\begin{array}{l} k\left(i,j\right)=\{\begin{array}{c} 1\;if\;the\;distance\;between\;localization\;i\;and\;j\;is\\ \;less\;than\;d\\ 0\;otherwise\\ 0\;if\;the\;localizations\;i\;and\;j\;are\;identical \end{array} \end{array}$$

For efficient computing on large datasets, we limited the number of test localizations to a subset with typically 500 localizations. For comparison with experimental data, we generated data sets with random localizations according (i) to a Poisson point process, and (ii) to a Neyman-Scott point process (Neyman and Scott, 1952). The Poisson process yields a data set of complete spatial randomness, whereas the Neyman-Scott process yields a data set with spatially Poisson-distributed parent events. Each parent event provides a set of offspring events with a Poisson distributed number of members, on average 5 (equal to the average number of localizations per fluorophore obtained experimentally from diluted reference samples). The offspring spatial coordinates are 2D Gauss distributed around each parent event with a standard deviation equal to the localization precision of 8 nm. We generated data sets with an overall localization density equal to the densities of experimental data. Simulations and statistical analysis of five cells in each data set was carried out using Wolfram Mathematica 10.4.1.

## Commentary

### Comparison with other methods

During the last decades, fluorescence microscopy has allowed the direct observation of cellular processes in a relatively non-invasive fashion with high molecular specificity and temporal resolution. However, due to the wave nature of light the spatial resolution is limited to approximately half the wavelength of the light in the imaging plane (Abbe, 1873). Recently, super-resolution microscopy methods have circumvented this problem improving the optical resolution substantially. Localization microscopy exhibits the highest spatial resolution of less than 20 nm, as compared to other super-resolution techniques such as STED (Klar et al., 2000) or structural illumination microscopy (SIM) (Gustafsson, 2000). Moreover, due to their single molecule sensitivity, localization microscopy can potentially provide quantitative information about the spatial organization of proteins, as well as the number of molecules residing inside and outside of subcellular compartments including PM nanodomains involved in different cell functions. For example, PALM and *d*STORM, in combination with genetically encoded fluorescent photactivable proteins and immunochemistry, respectively, demonstrated nanocluster organization of synaptic proteins (Bar-On et al., 2012; Ehmann et al., 2014), membrane receptors involved in cell growth, proliferation and differentiation (Gao et al., 2015), tumor necrosis (Fricke et al., 2014), or related to the immunological response (Williamson et al., 2011; Rossy et al., 2013). Comparative studies have also proven PM heterogeneity depending on protein membrane anchor types including the transmembrane protein Lat, the lipid-anchored protein Lyn, the vesicular stomatitis viral glycoprotein VSVG, and GPI anchored proteins (Sengupta et al., 2011, 2013). However, all these studies were restricted to a limited number of proteins at a given time and thus, it became obvious that a more general approach for visualizing simultaneously a large population of membrane proteins is required to inspect the global distribution of PM proteins at the nanoscale. Moreover, fluorescent staining with antibodies and genetically encoded fluorescent proteins can induce artificial clustering of membrane proteins (Tanaka et al., 2010; Magenau et al., 2015) and limit the localization precision due to their relatively large size, especially in high density labeled samples. Metabolic labeling fills both gaps by introducing small bioorthogonal chemical groups such as azides into newly synthetized proteins.

Metabolic labeling has been used during the last decade to visualize newly synthetized proteins with standard fluorescent microscopy in cultured cells, tissues, and living animals. The advantage of this staining strategy is two-fold. First, labeling proteins with small and bioorthogonal chemical handles either by co-translational incorporation of unnatural amino acids or by post-translationally modification with non-natural monosaccharides minimizes perturbation of proteins and likely resembles physiological conditions. Second, metabolic labeling constitutes a unique tool to visualize spatial patterns of wide parts of the proteome. Whereas, immunochemistry and genetically encoded fluorescent are useful to visualize one specific protein, metabolic labeling allows to stain simultaneously newly synthesized proteins in a less specific way. Because the azido amino acid L-azidohomoalanine (L-AHA) replaces endogenous methionine, all proteins containing natively at least a single methionine are prompted to be labeled. On the other hand, the peracetylated azido sugar N-azidoacetylgalactosamine (Ac_4_GalNAz) is incorporated into specific subtypes of glycans such as mucin-type O-linked glycans and O-GlcNAc-modified glycoproteins (Laughlin and Bertozzi, 2009a). Further identification of which proteins incorporated successfully L-AHA or Ac_4_GalNAz has been achieved using alkyne affinity-tags (e.g., biotin-FLAG-alkyne tag) instead of alkyne fluorophores, in combination with proteomics studies (Dieterich et al., 2006; Laughlin et al., 2006). It is important to remark that the incorporation of L-AHA and Ac_4_GalNAz into PM proteins occurs during protein translation and post-translational glycosylation before they are delivered to the cell membrane. Therefore, different metabolic labeling conditions (e.g., changes in incubation time or concentration of the azido surrogates) can be used to study not only the spatial but also the temporal organization of newly synthesized proteins and glycans as shown previously by standard live-cell fluorescence microscopy (Baskin et al., 2007; Beatty and Tirrell, 2008; Laughlin et al., 2008; Laughlin and Bertozzi, 2009b; Dieterich et al., 2010).

When combined with super-resolution microscopy, metabolic labeling allows to inspect the overall distribution of membrane proteins at the nanoscale. This has recently been proven by STED and *d*STORM imaging of membrane proteins containing unnatural amino acids and azido sugars respectively (Letschert et al., 2014; Saka et al., 2014). Although both techniques provide images with substantially enhanced spatial resolution, due to their peculiarities, they exhibit unique advantages and limitations. For example, *d*STORM exhibits better spatial resolution than STED and has the potential to quantify molecular densities of membrane components as well as their spatial distributions. However, due to fluorophore photoswitching kinetics, the necessity of high photon yields, and slow camera frame rates, image acquisition typically requires few minutes (van de Linde et al., 2011). On the other hand, STED achieves much higher temporal resolution and therefore it is more suitable for dynamic studies. Remarkably, STED combined with fluorescence correlation spectroscopy (STED-FCS), where very small areas are scanned at frequencies in the order of a few kHz, can be used to measure diffusion dynamics of membrane proteins and lipids demonstrating molecular confinement with both high spatial and high temporal resolution (Eggeling et al., 2009; Saka et al., 2014).

### Critical parameters, limitations, and perspectives

**Figure 2:**
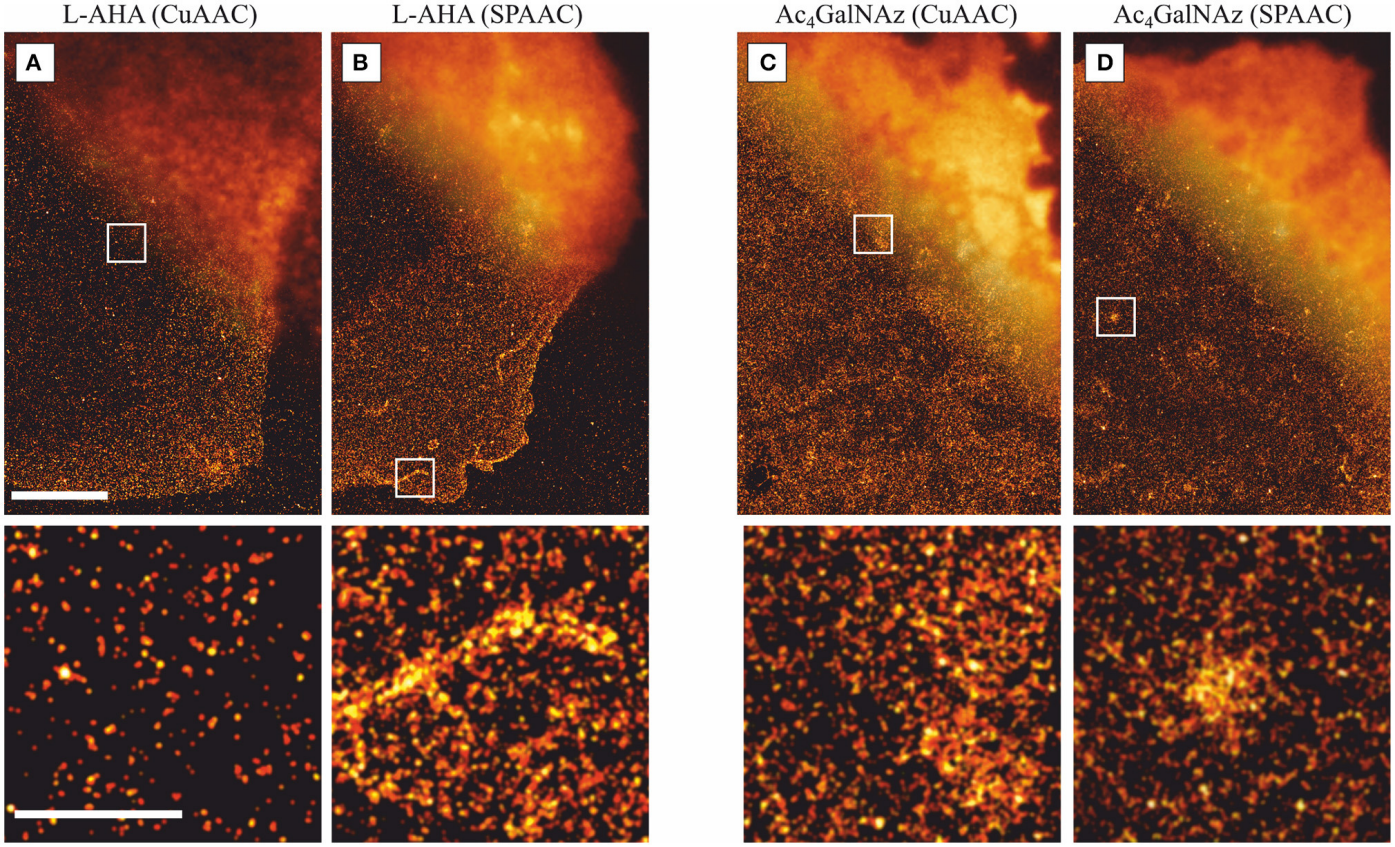
**Comparison of ***d***STORM images with standard fluorescent microscopy**. Representative *d*STORM and overlaid standard fluorescence images (upper right corner) of PM proteins at the basal membrane stained via **(A)** L-AHA (CuAAC), **(B)** L-AHA (SPAAC), **(C)** Ac_4_GalNAz (CuAAC), and **(D)** Ac_4_GalNAz (SPAAC). Comparison of L-AHA and Ac_4_GalNAz stained via copper-catalyzed (CuACC) and copper-free (SPAAC) show no significant differences, indicating that the presence of copper ions or THPTA do not affect the distribution of PM components. For the four staining schemes depicted, 2D projected structures lead to spatial inhomogeneties as highlighted in the lower panels, e.g., **(A)** one fold membrane under the nucleus, **(B)** two-fold membrane structure within the lamellipodia plus one filopodia, **(C)** membrane ruffles, and **(D)** projection of a vesicle located in close proximity to the plasma membrane. All images were acquired under TIRF illumination, reconstructed with a minimum localization intensity threshold of 1000 photons, and a pixel size of 10 nm. Scale bars are 5 μm (upper panels) and 1 μm (lower panels).

The conditions presented in the given protocols constitute a robust recipe to stain and visualize large populations of PM proteins and glycans with super-resolution localization microscopy (Figure 2). Nevertheless, critical aspects, as well as limitations and future perspectives, with regard to obtain reliable quantitative data and avoid artifacts are shown in the next subsections. First, we highlight potential artifacts of *d*STORM as well as the inherent problem of 2D super-resolution images due to projections of 3D structures such as membrane ruffling, filopodia, overlapping membranes, and vesicles located in close proximity to the PM. Then, we compare the fluorescence staining efficiency achieved by copper-catalyzed and copper-free click chemistry reactions for fixed metabolic labeling conditions. Finally, we show how quantitative information about the distribution of PM components can be percolated from *d*STORM data using statistical spatial analysis approaches, such as pair-correlation and Ripley's K functions.

### Artifacts and 2D projections of 3D structures in *d*STORM imaging.

The intrinsic features of localization microscopy, i.e., reconstruction of super-resolution images from localization of single molecules, determine its accuracy, and reliability. The precision of position determination of single and well isolated fluorescent emitters is mainly determined by the number of collected photons, the signal-to-noise ratio, and the accuracy of the algorithm implemented in the localization software used to fit the point-spread-function (PSF) of detected fluorophores (Thompson et al., 2002; Mortensen et al., 2010; Sage et al., 2015). In contrast, other considerations must be taken into account to reconstruct reliable super-resolution images. For example, overlapping PSFs of multiple fluorophores residing in their on-state simultaneously within the same diffraction-limited area must be prevented, except specialized algorithms capable of fitting multiple emitters PSFs are used (Holden et al., 2011; Zhu et al., 2012), to avoid incorrect localizations and ensure artifact-free images reconstruction (van de Linde et al., 2010; Sauer, 2013; van de Linde and Sauer, 2014; Burgert et al., 2015). As a rule of thumb to avoid PSFs overlapping and ensure reliable spot finding and fitting, the density of fluorescent emitters has to be kept below 0.2 spots per μm^2^ (Wolter et al., 2011). Therefore, appropriate measurement conditions in *d*STORM imaging such as laser irradiation intensities high enough to transfer the majority of organic dyes to long-living off states as well as suitable buffer compositions are required to guarantee good image quality.

**Figure 3:**
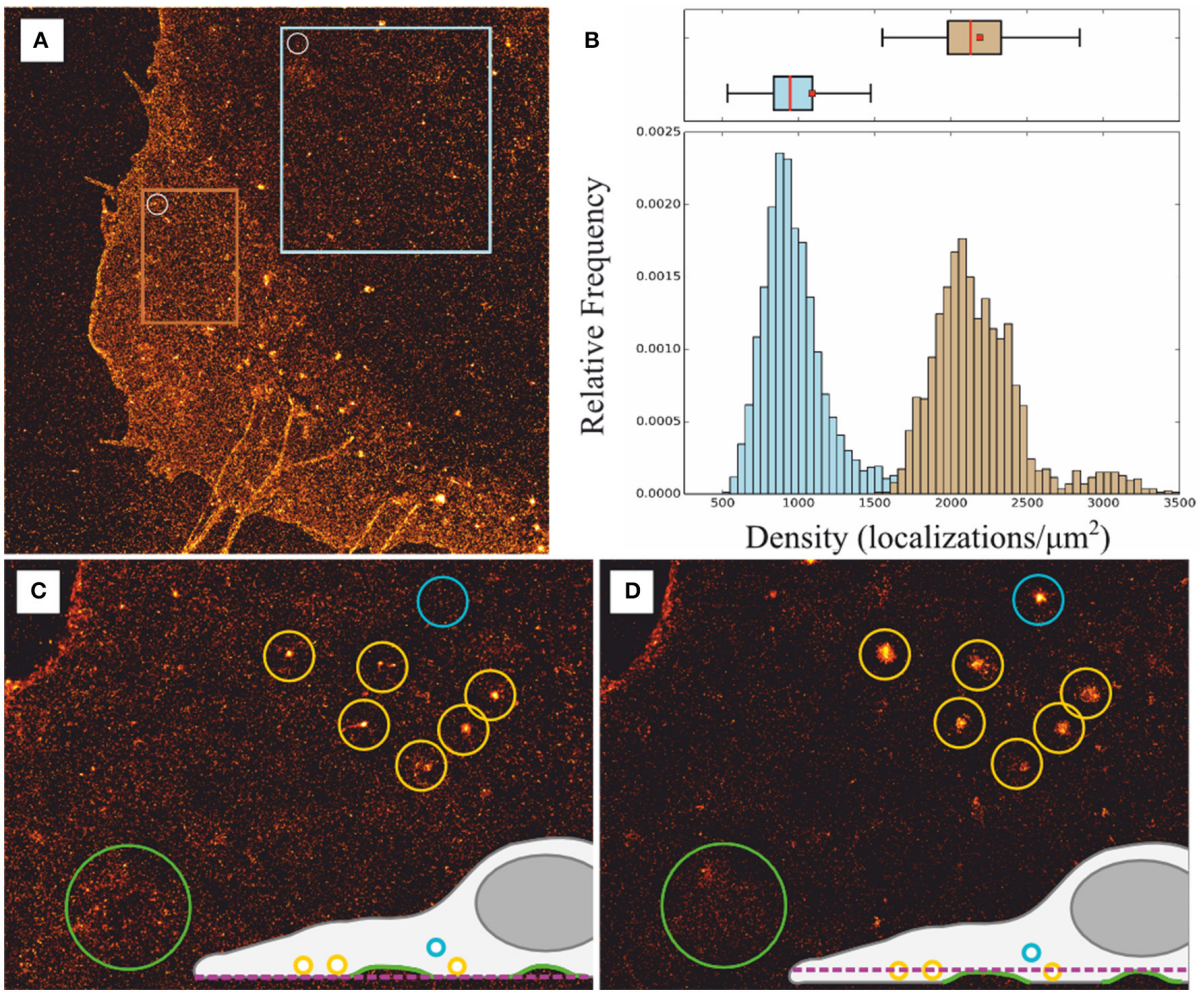
**Effect of two-dimensional projections of membrane structures. (A)** *d*STORM image of PM proteins metabolically labeled with L-AHA showing overlapping membranes, vesicle-like structures, and filipodia. **(B)** Sliding window analysis to estimate PM content (white circle in **(A):** diameter = 1 μm, step = 100 nm) lead to median values of 884 localizations per μm^2^ within a region under the nucleus, i.e., single membrane structure blue square in **(A)**, and 2130 localizations per μm^2^ within the lamellipodia, i.e., two-fold overlapping membranes orange square in **(A)**. Box plot: red bar = median, box = 25th and 75th percentile, □ = mean. **(C,D)** Consecutive images with focal planes slightly shifted (0.5–1 μm) into the cytosol reveal artificial cluster structures generated due to vesicle-like structures located above the plasma membrane blue and yellow circles as well as inhomogeneities due to membrane ruffles green circle; adapted from Burgert et al. (2015).

Besides the aforementioned experimental traits of *d*STORM, inherent problems and limitations appear when studying membrane components with 2D localization microscopy. Without 3D information the ability to extract unbiased information about PM can be error prone. The existence of Z-projections of inherent cell membrane structures such as invaginations and vesicle-like structures, including fluorophore-filled endosomes in contact with or located near the PM, as well as overlapping membranes in the lamellipodia, might distort severely the quantitative analysis and interpretation of super-resolution images. For example, a sliding window analysis applied to *d*STORM images of PM under the nucleus reveals half of the localization density compared to lamellipodia indicating a two-fold membrane structure (Figures 3A,B). Furthermore, circular clusters with apparent sizes ranging from a few tens to a few hundred nanometers can be visually identified from more homogeneous distributions, however it is difficult to discern weather they represent nanodomains enriched in membrane proteins or projections from fluorophore-filled vesicles in close proximity to the membrane. Whereas, a 3D-*d*STORM measurement would reduce any information bias on PM organization due to vertical projections, instrumentation, and implementation for 3D-*d*STORM is more complex and expensive compared to 2D-*d*STORM, and they usually achieve a lower axial than lateral resolution (Klein et al., 2014). In contrast, consecutive imaging of the same cell with slightly shifted focal planes above the feature of interest constitutes a fast control to determine the two-dimensional projection contribution from inherent 3D structures as shown in Figures 3C,D for vesicle-like structures located right above the plasma membrane (yellow circles) or further up (blue circle), and membrane ruffles (green circle) (Burgert et al., 2015).

### Optimal staining efficiencies by copper-catalyzed and copper-free click-chemistry.

The first step of any fluorescent microscopy technique is the efficient staining of the protein of interest with a fluorophore. Moreover, in localization microscopy higher staining efficiencies, reflected as higher labeling densities, affects the maximum resolution in localization microscopy (Sauer, 2013). Whereas, imaging resolution is usually defined as the minimal resolvable distance between two emitters, the extractable structural information is also related to the sampling frequency, i.e., fluorophore labeling density, as described by the Nyquist-Shannon theorem (Shannon, 1949). In essence, the theorem states that the sampling interval, i.e., the mean distance between neighboring localized fluorophores, must be at least twice as fine as the structural details to be resolved. Therefore, higher labeling densities prevent under sampling and improve spatial resolution.

**Figure 4:**
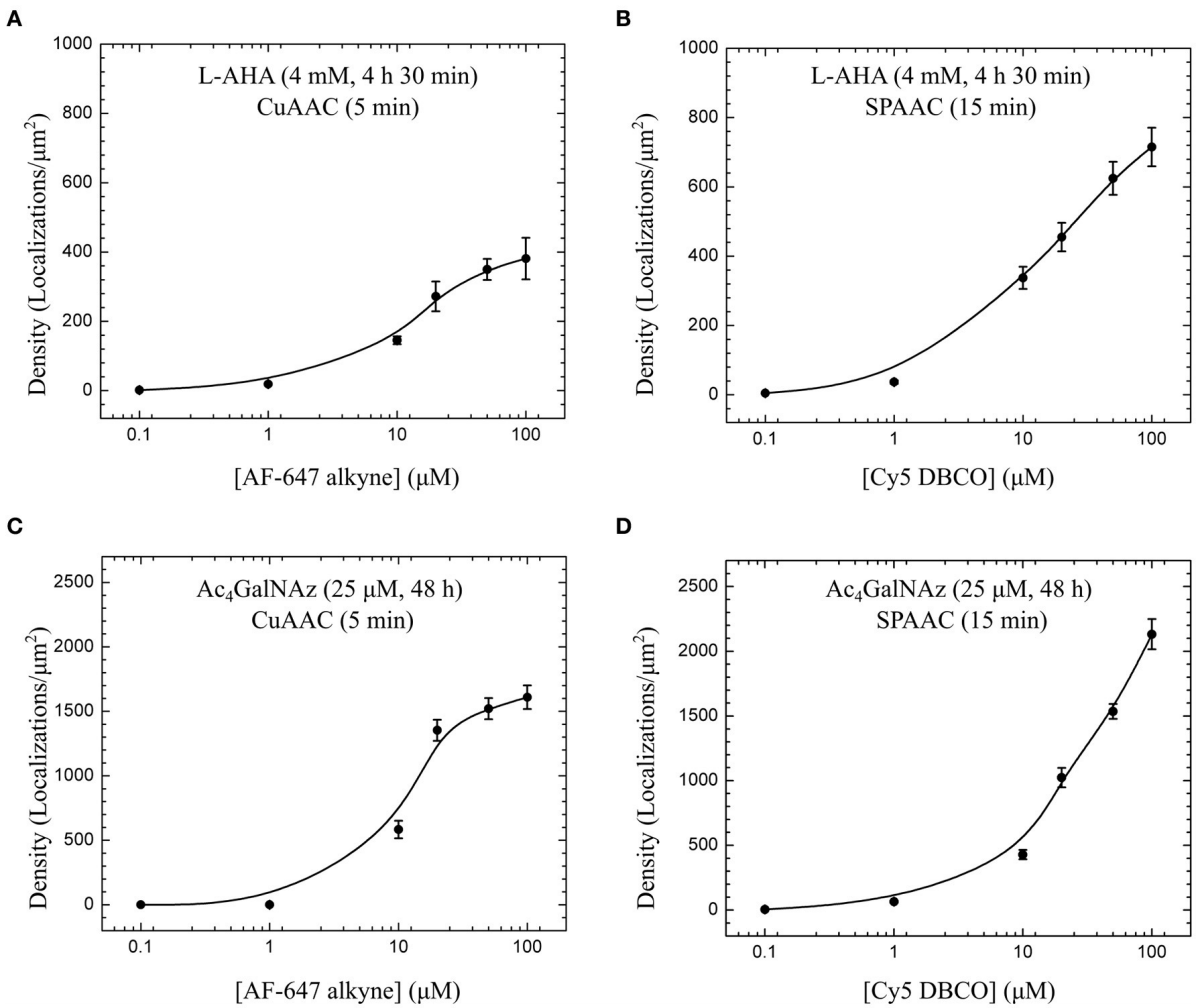
**Labeling efficiency of copper-catalyzed (CuAAC) and copper-free azide-alkyne cycloadditions (SPAAC)**. Fluorophore titration for the same metabolic labeling conditions, i.e., 4 mM L-AHA during 4 h 30 min **(A,B)**, and 25 μM Ac_4_GalNAz during 48 h **(C,D)**, show optimal staining efficiency with AF-647 alkyne and Cy5 DBCO in the range of 20 to 50 μM for 5 min CuAAC and 15 min SPAAC reactions. For each cell, detected localizations were first obtained with a sliding window analysis (diameter = 1 μm, step = 100 nm) applied to big areas defined at bottom plasma membrane under the cell nucleus as described in Figure 3B. Plotted values and error bars represent median and SE of several cells imaged and analyzed for each fluorophore concentration [**(A)** 7–10 cells, **(B)** 8–15 cells, **(C)** 7–8 cells, and **(D)** 12–16 cells].

The conditions given here for click chemistry staining of membrane proteins and glycoconjugates lead to maximum labeling densities ranging from 400 to 2000 localizations per μm^2^ (Figure 4). For the four bioconjugated systems inspected, we observed that fluorophore concentrations around 20–50 μM are required to maximize fluorescent signal. Moreover, copper-free strain-promoted azide-alkyne cycloaddition (SPAAC) is equally efficient as CuAAC to stain Ac_4_GalNAz-derived glycoconjugates, and two-fold better to detect membrane proteins containing AHA. Thus, optimal conditions for click chemistry can also be achieved in absence of copper avoiding toxicity effects and simplifying the protocol.

### Quantitative analysis with *d*STORM

**Figure 5:**
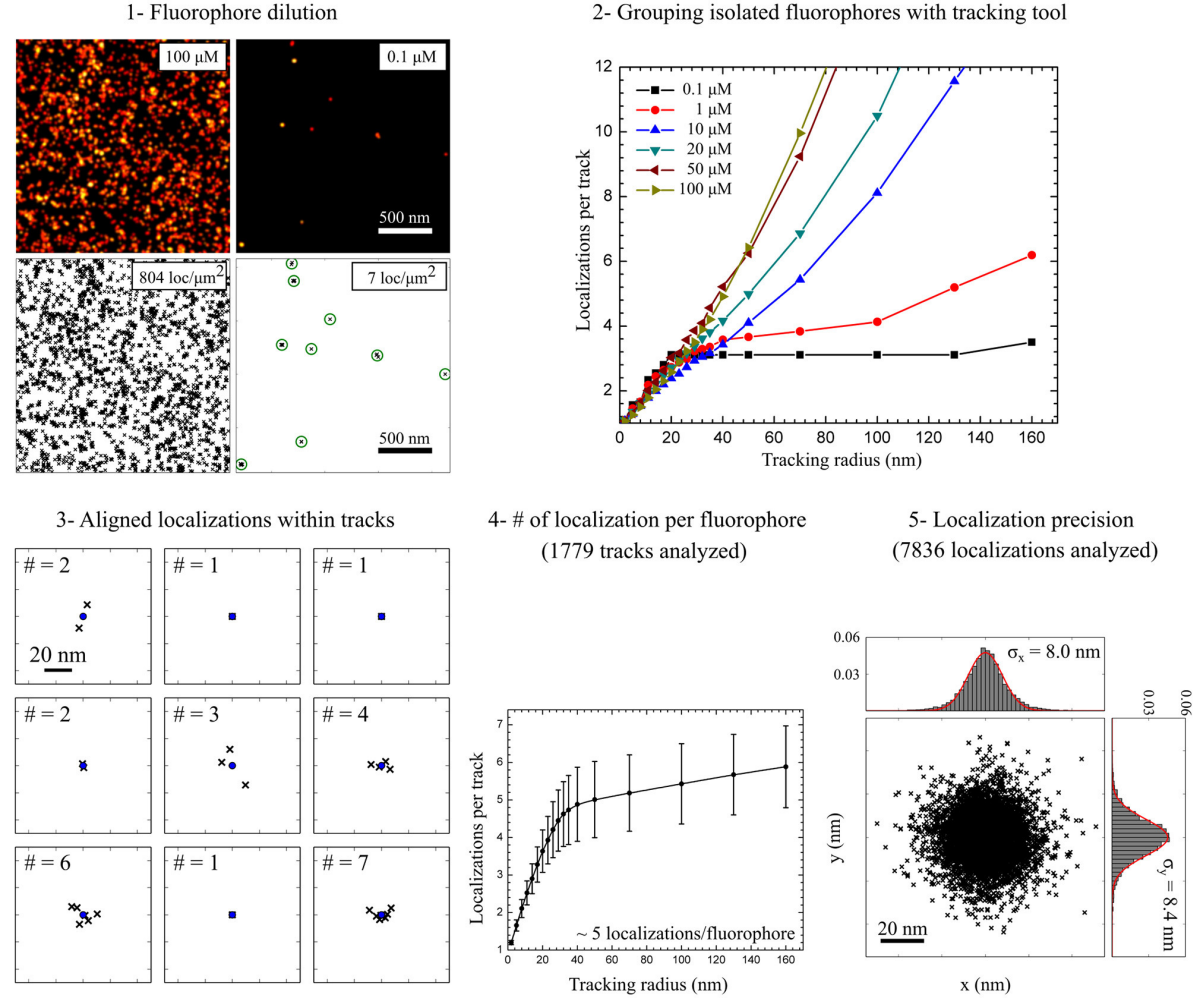
**Estimation of molecular densities and experimental localization precision**. (1) Fluorophore dilution (<0.1 μM) leads to very low localization densities (<20 localizations per μm^2^) allowing the detection of well isolated fluorophores. (2) Grouping localizations from isolated fluorophores was performed with a tracking algorithm. To confirm the detection of isolated fluorophores the tracking radius was varied from 1 to 160 nm for different fluorophore concentrations. (3) Localizations within tracks detected using a tracking radius = 50 nm aligned to the center of mass of each track. (4) For diluted samples the saturation level (tracking radius = 50 nm) indicates the number of localization per track, i.e., the number of localizations per isolated fluorophore. (5) Aligned localizations are used to estimate the experimental localization precision by fitting X and Y projections of the probability density function to a Gauss function.

In *d*STORM measurements, localization densities in a certain area of the sample can be directly calculated from the coordinate lists exported by the localization software. Whereas, the number of localizations per unit area can be used to estimate the staining efficiency for different labeling conditions, it only provides relative information on the detected numbers of membrane proteins present. Since organic dyes undergo several photoswitching cycles during a *d*STORM measurement, counting molecular numbers with localization microscopy requires further correction for multiple detections of the same molecule. The typical number of localizations recorded per fluorophore under the same optical and chemical conditions can be determined in diluted samples (Figure 5). If the blinking of isolated spots can be unequivocally assigned to single fluorophores, a conversion factor can be extracted to estimate the detected number of labeled membrane proteins (Table 1). For example, we estimate the density of PM proteins labeled with AHA during 4 h 30 min to be approximately ~50 μm^−2^ and ~125 μm^−2^ when stained via CuAAC and SPAAC respectively. On the other hand, we detected higher densities of glycans, in the range of ~345 μm^−2^ and ~280 μm^−2^, metabolic labeled with Ac_4_GalNAz during 48 h. It is important to mention that dividing the number of localizations in a region of interest by the average number of localizations detected per isolated fluorophore in reference experiments represents only an average correction value. To prevent over-counting effects in highly dense sample areas, more sophisticated methods based on the temporal and spatial fingerprint of single fluorophore blinking, such as off-time gap (Zhao et al., 2014) and pair correlation function analysis (PCF) (Veatch et al., 2012; Sengupta et al., 2013), can be applied.

**Table 1:** **Quantification of molecular density and experimental localization precision**.

	**Localization density^a^ (loc/μm^2^)**	**Conversion factor^b^ (loc/fluorophore)**	**Molecular density (fluorophore/μm^2^)^c^**	**σ_*x*_ (nm)^d^** **σ_*y*_ (nm)^e^**
AHA (CuAAC)	350 ± 30	6.7 ± 1.1	52 ± 13	8.7 ± 0.1 8.9 ± 0.1
AHA (SPAAC)	625 ± 48	5.0 ± 1.0	125 ± 35	8.0 ± 0.1 8.4 ± 0.1
Ac_4_GalNAz (CuAAC)	1520 ± 82	4.4 ± 1.4	345 ± 128	8.6 ± 0.1 9.9 ± 0.1
Ac_4_GalNAz (SPAAC)	1536 ± 58	5.5 ± 1.0	279 ± 61	8.2 ± 0.1 8.4 ± 0.1

a *Localization densities reflect median values calculated with a sliding window (diameter = 1 μm step = 100 nm) in regions under the cell nucleus to avoid overlapping membranes as shown in Figure 3. Data presented correspond to 50 μM fluorophore concentration, i.e., AF-647 alkyne for 5 min CuACC staining and Cy5 DBCO for 15 min SPAAC staining*.

b *Number of localizations per fluorophore obtained in diluted samples as described in Figure 4 for 0.1 μM fluorophore concentrations*.

c *Detected molecular densities calculated from localization densities divided by localizations per fluorophore*.

d, e *Standard deviations obtained from Gauss function fits of the probability density functions calculated from aligned localizations as described in Figure 4*.

Beyond density determination, coordinate lists obtained by localization microscopy can be used advantageously to inspect spatial distributions of membrane proteins. Analysis based on pair-correlation function (PCF) (Veatch et al., 2012; Sengupta et al., 2013) or nearest-neighbor based algorithms (including *Ripley's K* function) (Owen et al., 2012) can indicate weather proteins are more aggregated forming clusters or more dispersed than they were under a distribution of complete spatial randomness. All analysis routines need to take into account local self-clustering induced by single fluorophore blinking. Moreover, quantitative estimation of cluster size and densities can be difficult to extract without prior biological knowledge (Coltharp et al., 2014). Nevertheless, comparison with simulated spatial distributions mimicking experimental data can alleviate these problems and avoid miss-interpretations (Kiskowski et al., 2009; Veatch et al., 2012; Letschert et al., 2014). Finally, clustering algorithms, such as K-Means, DBSCAN, and polygon-based tessellation methods, have been used for morphological analysis of membrane proteins (Bar-On et al., 2012; Ehmann et al., 2014; Löschberger et al., 2014; Levet et al., 2015; Andronov et al., 2016). In contrast to pair-correlation and nearest-neighbor based algorithms, these methods rely on segmentation of the super-resolution image and thus the size and shape of each cluster, as well as their XY position, can be directly visualized.

To characterize the spatial distribution of PM components, we calculated Ripley's h functions from experimental data and two different sets of simulated spatial patterns. In particular, we simulated XY coordinates according to (i) a Poisson process and (ii) a Neyman-Scott process within 5 × 5 μm^2^ with similar density as the number of localizations per μm^2^ obtained from *d*STORM images. Whereas, a Poisson process resembles complete spatial randomness, it lacks to mimic individual fluorophore blinking inherent to *d*STORM measurements. In contrast, data sets simulated according to the Neyman-Scott process (Neyman and Scott, 1952) account photoswitching cycles from single fluorophores by including Gauss distributed offspring events around each parent position. Number of the offspring events and the standard deviation of the Gauss distribution (σ) where set from experimental data, i.e., on average ~5 blinks per fluorophore and experimental localization precision ~8 nm, respectively.

**Figure 6:**
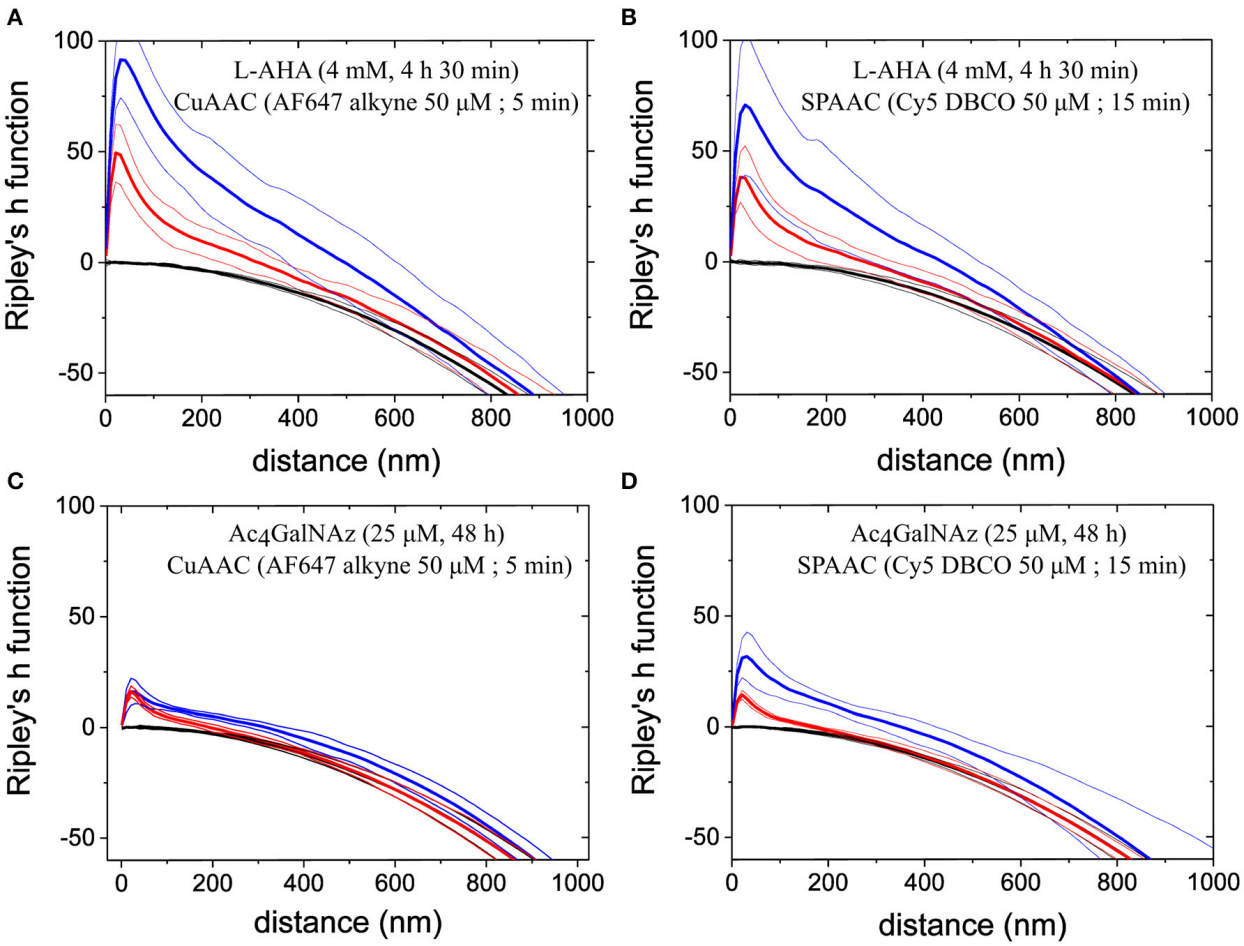
**Spatial distribution analysis by Ripley's h function**. The data show Ripley's h functions computed from experimental data (blue lines) of PM proteins stained via **(A)** L-AHA (CuAAC), **(B)** L-AHA (SPAAC), **(C)** Ac_4_GalNAz (CuAAC), and **(D)** Ac_4_GalNAz (SPAAC). Plotted curves represent mean values (thick lines) together with 95% confidence intervals (thin) over 5 regions in total (5 × 5 μm^2^ size) from independent cells, which appeared rather homogeneous by visual inspection. For comparison Ripley's function was computed from two simulated random point process, i.e., Neyman-Scott process (red lines) and Poisson point process (black lines). Simulation parameters, such as process intensity, average of offspring events, and spatial distribution around their parent event, where chosen to mimic localization density, photoswitching cycles, and localization precision obtained experimentally. The peak observed on short length scales for Neyman-Scott process and experimental data indicates artificial clustering due to repeated localizations from identical fluorophores within a Gauss distributions equal to localization precision, i.e., standard deviation ~8 nm. For all four staining schemes presented, Ripley's h functions show further clustering on longer length scales but more pronounced for L-AHA samples.

Ripley's k function reveals possible combinations of homogeneous distributions on large scales and clustering on small scales (e.g., due to the repeated blinking of individual labels). Figure 6 shows direct comparison between experimental (blue line) and simulated data for a Poisson and Neyman-Scott process (black and red line respectively). For all the labeling schemes inspected, our data showed maximum clustering on a length scale similar to the estimated localization precision (i.e., d ~20–30 nm). Therefore, clustering might reflect single fluorophore photoswitching. Since the maximum value of Ripley's h function for a simulated Neyman-Scott process is close to that of experimental data, we conclude that single fluorophore blinking is the only significant clustering process on this length scale. In addition, all the data indicate a small but significant deviation from complete spatial randomness on length scales from 30 to 800 nm. It is important to note that there is no characteristic length scale above 30 nm for any clusters of a well-defined size that can be identified. The indicated deviations from complete spatial randomness can have their origin in the various PM deformations e.g., due to the onset of vesicle formation or membrane ruffling. Whereas, it is possible to find small areas with a distribution that perfectly resemble a Neyman-Scott process (with clusters originating only from single emitter blinking), Ripley's h function for data in areas of 5 × 5 μm^2^ in well-labeled cells under the nucleus (excluding double membrane contributions) typically appear as presented.

## Conclusions and remarks

We report a chemical reporter strategy, based on metabolic labeling and click chemistry, in combination with super-resolution imaging by *d*STORM to stain and visualize PM proteins and glycans. The labeling methodology results in staining efficiencies ranging from ~50 to ~350 fluorophore per μm^2^ depending on the labeling scheme used. Besides the estimation of PM protein content, our data show potential artifacts in super-resolution images due to 2D-projections of 3D-inherent cell structures. For example, overlapping membranes lead to overestimation of protein content, and vesicle-like structures located in closed proximity to the cell membrane appear as protein clusters and, thus, can potentially result in false interpretation of PM organization. Consecutive imaging with slightly shifted focal planes below and above the structure of interest can be used to reveal the contribution of 3D structures as two-dimensional projections. Furthermore, statistical analysis based on Ripley's function combined with point pattern simulations, can be used to identify deviations from complete spatial randomness. Our data clearly show artificial clustering due to fluorophore photoswitching at length scales related to the experimental localization precision (i.e., ~20–30 nm). Ripley's analysis also indicates a small deviation from spatial randomness at larger scales (e.g., ~30–800 nm). However, whereas these deviations from randomness might reflect some spatial organization of PM proteins at the nanoscale, their origin due to membrane modulations and ruffles, or the onset of vesicle formation cannot be completely excluded.

Finally, the examples presented here where performed at fixed metabolic conditions to incorporate azide groups in newly synthesized proteins. Experimental designs varying concentration and incubation time of metabolic surrogates combined with drug treatments can be used to study how fast proteins are delivered and trafficked from the cytosol to the plasma membrane. Reversibly, proteins can be followed after live cell staining to study membrane turnover involving different endocytic pathways. All in all, click chemistry constitutes a powerful tool to study PM composition at the molecular level as well as its dynamic organization. Moreover, the synthesis of new bioorthogonal molecules as well as their commercial availability will expand the applicability and usability of this methodology.

## Author contributions

PM and MS designed the experiments. PM and SL performed the experiments. PM and SD analyzed the data. All authors discussed results and contributed to the manuscript.

### Conflict of interest statement

The authors declare that the research was conducted in the absence of any commercial or financial relationships that could be construed as a potential conflict of interest.

## Supplementary material

Figure S1 **Click chemistry staining specificity. (A)** To evaluate non-specific signal, control cells were incubated with AHA in the presence of 40 μM anisomycin, a protein synthesis inhibitor, and subsequently stained via CuAAC or SPAAC with 50 μM of Alexa Fluor 647 alkyne for 5 min or Cy5 DBCO for 15 min respectively. **(B)** In the case of azido sugar, control cells were incubated in absence of Ac_4_GalNAz and subsequently stained via CuAAC or SPAAC with 20 μM of AF 647 alkyne for 5 min or Cy5 DBCO for 15 min respectively. All controls showed relatively low background of ~19, 42, 10, and 20 localizations per μm^2^ for L-AHA (CuAAC), L-AHA (SPAAC), Ac_4_GalNAz (CuAAC), and Ac_4_GalNAz (SPAAC) respectively. Values and error bars represent median and SE of localization densities obtained with sliding window analysis under the nucleus (*N* = 7 cells in all cases).
