# Supplementary figures and images for: Super-Resolution Imaging of Plasma Membrane Proteins with Click Chemistry
Source: Front Cell Dev Biol. 2016 Sep 9;4:98. doi: 10.3389/fcell.2016.00098 (PMC5016519; doi:10.3389/fcell.2016.00098)

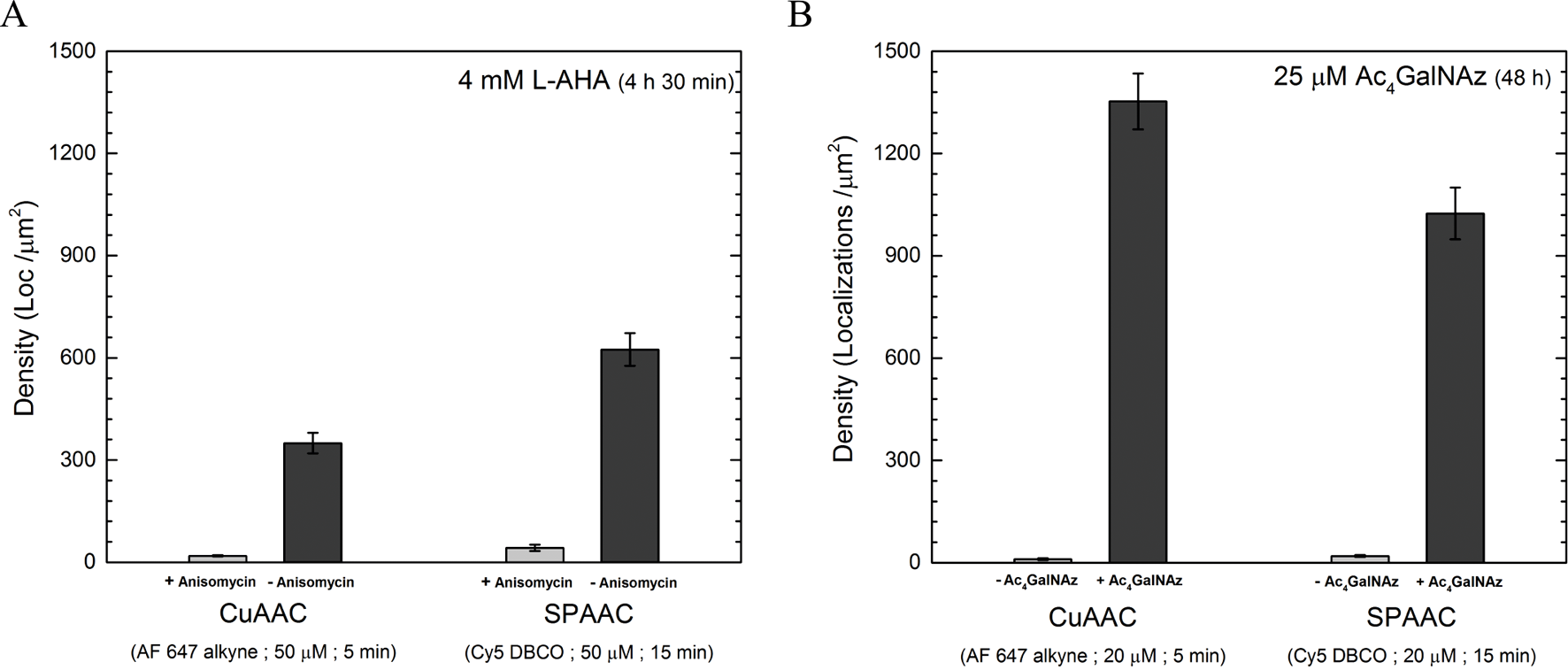

Supplement: Figure S1 — Click chemistry staining specificity. (A) To evaluate non-specific signal, control cells were incubated with AHA in the presence of 40 μM anisomycin, a protein synthesis inhibitor, and subsequently stained via CuAAC or SPAAC with 50 μM of Alexa Fluor 647 alkyne for 5 min or Cy5 DBCO for 15 min respectively. (B) In the case of azido sugar, control cells were incubated in absence of Ac4GalNAz and subsequently stained via CuAAC or SPAAC with 20 μM of AF 647 alkyne for 5 min or Cy5 DBCO for 15 min respectively. All controls showed relatively low background of ~19, 42, 10, and 20 localizations per μm2 for L-AHA (CuAAC), L-AHA (SPAAC), Ac4GalNAz (CuAAC), and Ac4GalNAz (SPAAC) respectively. Values and error bars represent median and SE of localization densities obtained with sliding window analysis under the nucleus (N = 7 cells in all cases). [file Image1.TIF]
